# Squamous cell carcinoma of the renal parenchyma presenting as hydronephrosis: a case report and review of the recent literature

**DOI:** 10.1186/s12894-020-00676-5

**Published:** 2020-07-20

**Authors:** Xirong Zhang, Yuanfeng Zhang, Chengguo Ge, Junyong Zhang, Peihe Liang

**Affiliations:** 1 Department of Urology, The Second Affiliated Hospital of Chongqing Medical University, Yuzhong, Chongqing, 400010 China; 2 Department of Urology, Jiangmen Central Hospital, Jiangmen, 529030 Guangdong China

**Keywords:** Kidney, Renal parenchyma, Squamous cell carcinoma, Hydronephrosis, Malignancy

## Abstract

**Background:**

Primary squamous cell carcinoma of the renal parenchyma is extremely rare, only 5 cases were reported.

**Case presentation:**

We probably report the fifth case of primary Squamous cell carcinoma (SCC) of the renal parenchyma in a 61-year-old female presenting with intermittent distending pain for 2 months. Contrast-enhanced computed tomography (CECT) revealed hydronephrosis of the right kidney, but a tumor cannot be excluded completely. Finally, nephrectomy was performed, and histological analysis determined that the diagnosis was kidney parenchyma squamous cell carcinoma involving perinephric adipose tissue.

**Conclusions:**

The present case emphasizes that it is difficult to make an accurate preoperative diagnosis with the presentation of hidden malignancy, such as hydronephrosis.

## Background

Squamous cell carcinoma (SCC) of the renal pelvis is a rare neoplasm, accounting for only 0.5 to 0.8% of malignant renal tumors [1], SCC of the renal parenchyma is even less common. A review of the literature shows that only five cases of primary SCC of the renal parenchyma have been reported to date [2–6]. Here, we are reporting a case of primary SCC of the renal parenchyma, which was post-surgically diagnosed according to tissue pathology. A review of recent related literature is provided as well. The ethics committee of Chongqing Medical University has reviewed and approved the project and the Ethics board approval number is 2016–064.

## Case presentation

The patient is a 61-year-old female. After suffering from intermittent pain in the right flank region for 2 months she was referred to the urology department at an outside hospital. The patient was diagnosed with hydronephrosis of the right kidney and underwent a right ureteroscopy with placement of a double J stent. The patient was subsequently discharged***.*** After removal of the double J stent (which occurred 1 month following discharge), the patient presented intermittent colicky pain at the right flank region. Subsequently, the patient was referred to our department for further evaluation and treatment. The patient had reviewed and provided written consent to participate in the study. The ethics committee of Chongqing Medical University has reviewed and approved the project and the Ethics board approval number is 2016–064.

In our department, we asked about her medical history in detail. She denied having symptoms such as frequent micturition, urgent urination, odynuria, hematuria, dysuria, abdominal pain, fever, chills, nausea or vomiting. Her medical history included hypertension with blood pressure up to 160/100 mmHg. She had never taken any medication for hypertension. There was no history of prior radiation exposure or kidney stones. She denied prior smoking and alcohol consumption. Physical examination revealed mild right costovertebral angle tenderness, but was otherwise normal. We performed the following auxiliary checks: Her routine blood tests demonstrated a red blood cell count (RBC) of 4.12 × 10^12^/L, white blood cell count (WBC) of 10.75 × 10^9^/L, and a total number of platelets (PLT) of 332 × 10^9^/L. The erythrocyte sedimentation rate (ESR) was 70 mm/h. Procalcitonin (PCT) was 0.949 ng/mL (normal, < 0.50 ng/mL). Tuberculosis T cell detection and the Tuberculin Purified Protein Derivative (PPD) test were within the normal ranges. Her serum chemistry, routine urine tests, renal and liver function were within the normal ranges as well.

**Fig. 1:**
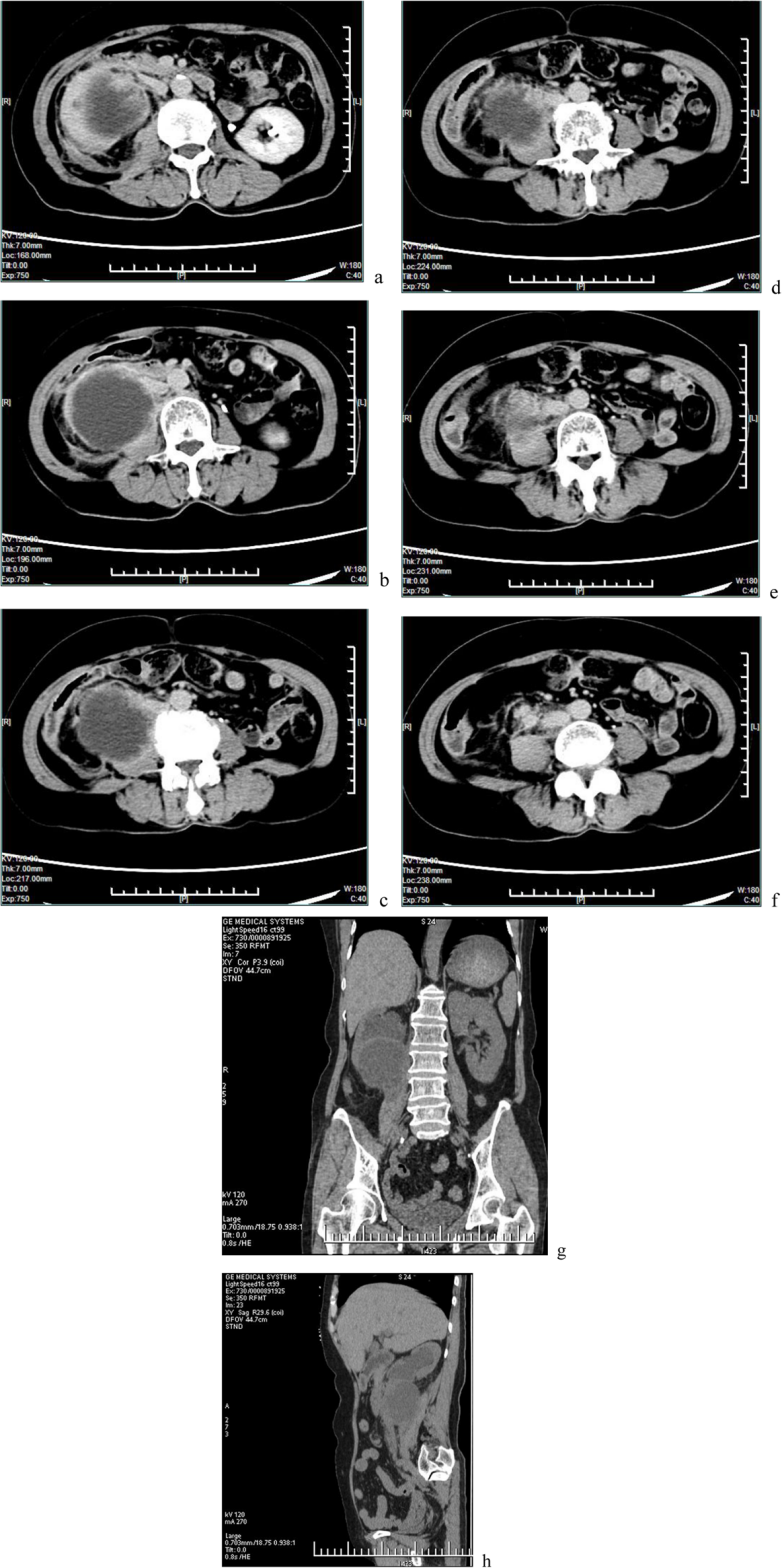
**a**-**c** Contrast-enhanced computed tomography showed marked hydronephrosis of the right kidney and some perinephric exudates. **d**-**f**. Contrast-enhanced computed tomography revealed right gross hydronephrosis and right perinephric chronic inflammation; in the lower pole of the right kidney, a tumor could not be completely excluded with mild to moderate enhancement. **g**,**h** Plain scan of coronal and sagittal images

Her chest X-ray was within the normal range, but ultrasonography of the urinary system revealed heavy hydronephrosis of the right kidney. Contrast-enhanced computed tomography (CECT) of the abdomen showed marked hydronephrosis of the right kidney (which presented as a cyst-like structure), and some perinephric exudates (Fig. 1a-c). Further CECT (Fig. 1d-f) and CT (coronal and sagittal, Fig. 1g,h) of the abdomen revealed right gross hydronephrosis with a markedly dilated cystic pelvicalyceal system and right perinephric chronic inflammation; a tumor could not be completely ruled out in the lower pole of the right kidney due to uneven renal parenchyma and mild to moderate enhancement in the nephritic phase. Renal radionuclide imaging revealed that the glomerular filtration rate (GFR) of the right kidney was 12.3 mL/min, GFR of the left kidney was 57.9 mL/min and the total GFR was 70.2 mL/min (normal GFR>64.8 mL/min). A careful study of the imaging ruled out the presence of any other systemic involvement. Infectious lesions were considered before surgery, but tumors could not be excluded. The patients and her family members refused to perform other further examinations.

**Fig. 2:**
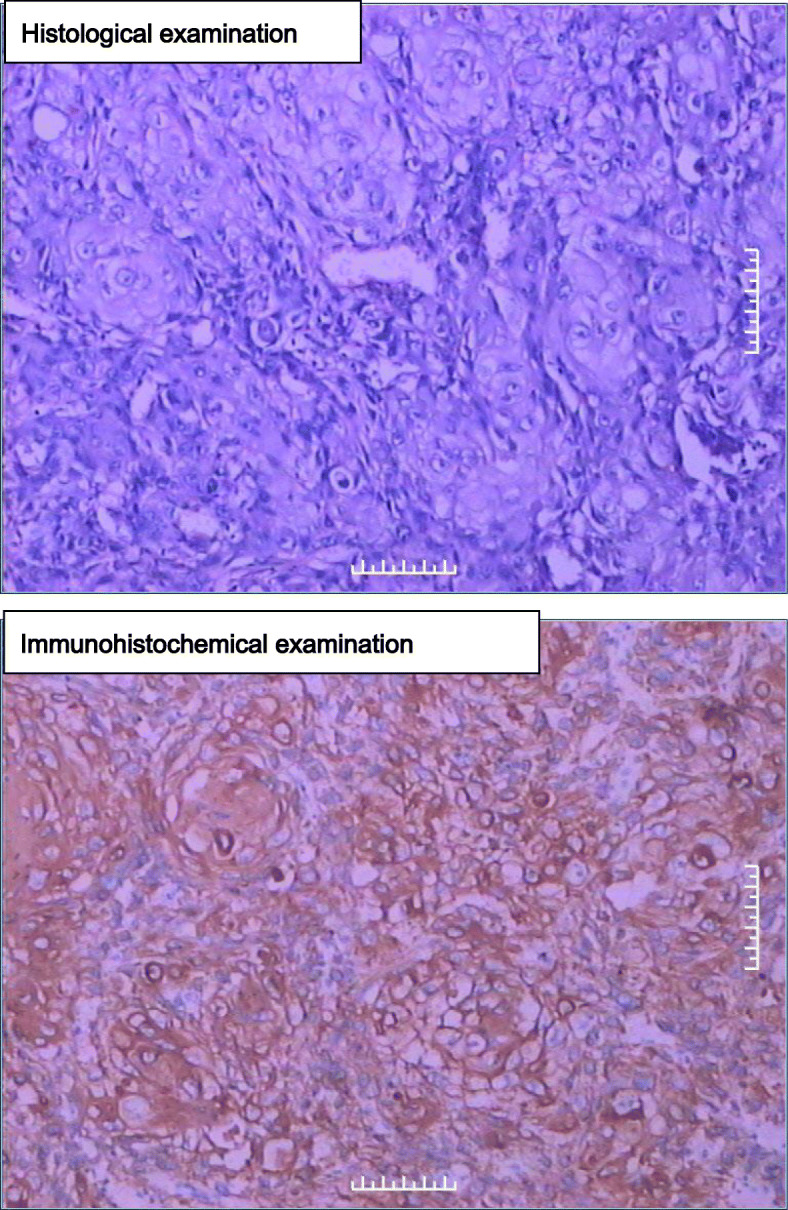
(upper) Histological examination, showing classical morphology of squamous carcinoma (HE staining, 200×). (Fig. 2 lower) immunohistochemistry, CK5/6 positive (200×)

**Fig. 3:**
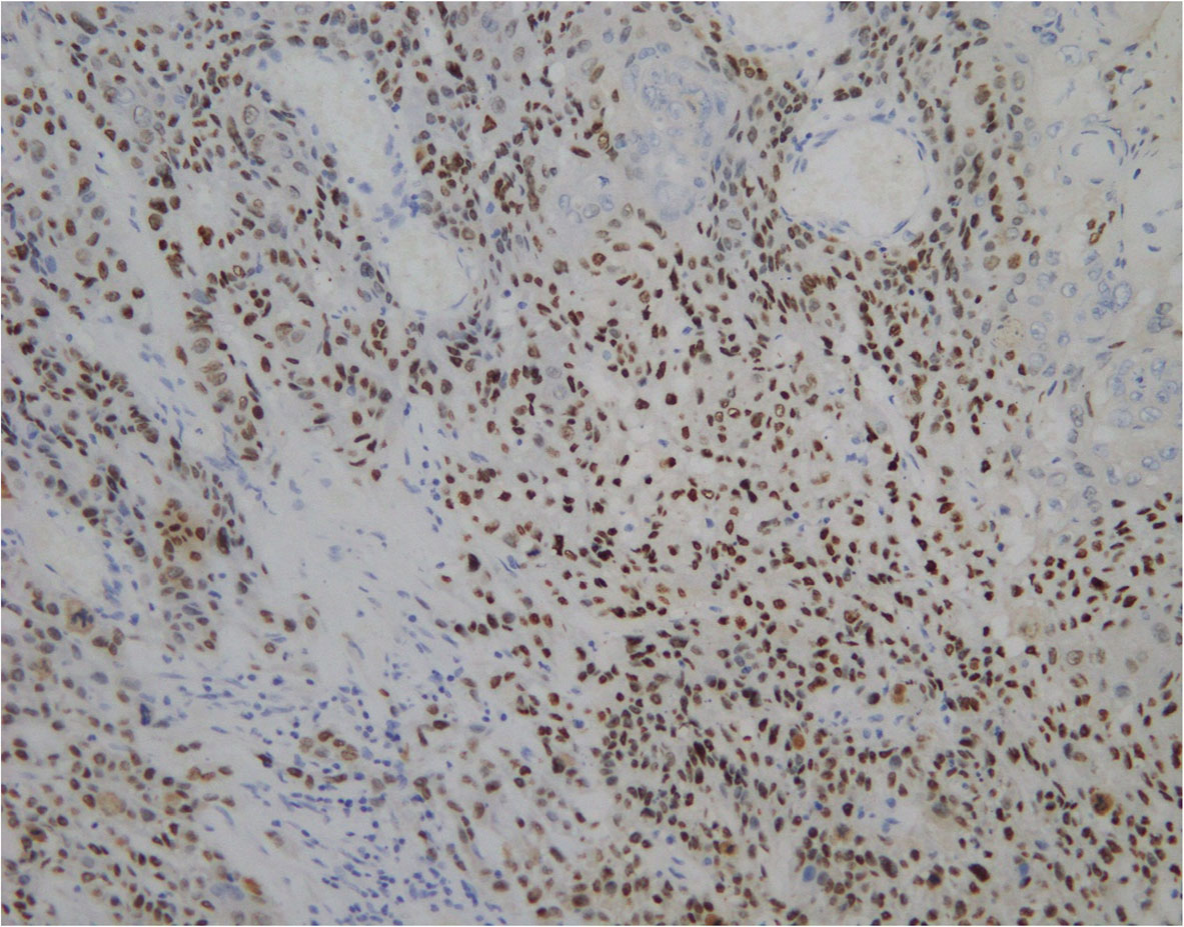
immunohistochemistry of p63, positive (+++) (200×)

Retroperitoneal laparoscopic radical nephrectomy was performed under general anesthesia. Severe perinephric adhesions were found during the procedure (especially the hilum), therefore lymph nodes were not subjected to lymphadenectomy. It can be seen that the resected kidney specimen presented an irregular and enlarged “skin capsule” shape. The thickness of the capsule wall was uneven, and the thickening was obvious in some areas, especially in the lower pole, but there was no round or oval solid mass with any clear boundary. The resected specimen was submitted to histopathology following surgery. The pathologist evaluated the right kidney (11.0 × 6.0 × 5.0 cm) with the upper ureter (0.8 × 0.6 cm). The bisected right kidney showed a grayish white cut surface and a markedly dilated cystic pelvis, incrassated parenchyma of the lower pole, and incrassated renal capsules. The histological examination revealed well-differentiated squamous cell carcinoma (SCC), displaying typical morphological features of SCC (including: epithelioid cell niche, mild to moderate heterotypic cells, visible keratin pearls and intercellular bridges). Immunohistochemistry results also supported this diagnosis. Normal glomeruli and tubule structures were also observed around the cancerous foci, involving perinephric adipose tissue, but not including the renal pelvis, renal vein, or ipsilateral ureter. There was no distant metastasis***.*** Therefore, a pathological stage of pT3aNxM0 was assigned. Immunohistochemistry showed EMA(−), CK(+++), HCK(+++), CK5/6(++), LCK(++), P63(+++), CK8(−), Ki67(++), 40% P53(+++), CerbB-2(−) (Figs. 2 and 3).

The patient refused additional treatment after surgery. She attended the phone call follow-up for 3 months, after which she was lost to follow-up. Written informed consent was obtained from the patient and her immediate family for the publication of the present study.

## Discussion and conclusions

SCC of the kidney is a rare clinical entity representing only 0.5 to 0.8% of malignant renal tumors [1]. SCC of the renal parenchyma is extremely rare when compared to SCC of the renal pelvis. No less than 15 literature references have reported SCC of the renal pelvis in the recent 5 years, and a current review of the literature could identify only 5 literature references concerning SCC of the renal parenchyma [2–7] (Table 1). Until recently, SCC of the renal pelvis was regarded as urothelium metaplasia [7–9], however, the histological origin and mechanism of renal parenchyma SCC remains unknown. It probably originates from convoluted tubules or undifferentiated stem cells of the renal mesenchyme. SCC of the kidney is frequently associated with urolithiasis and hydronephrosis [3, 5, 6]. The present patient had a history of hydronephrosis for 2 months and surgery was performed to relieve the hydronephrosis. Hydronephrosis is a predisposing factor resulting in squamous metaplasia, which may develop into SCC later on.

**Table 1:** Characteristics of the reported cases

Author	Sex	Age	Presentation	radiological feature	Treatment	Prognosis
Terada (2010) [2]	M	73	Hematuria and lumbago	A bladder tumor, a left ureter tumor, and a left kidney tumor.	Nephroureterectomy	Alive at 3 months after surgery
Kulshreshtha (2012) [3]	F	60	Significant weight loss of 3 months duration	A mass in the mid and lower pole of left kidney with areas of necrosis are notable, and retroperitoneal lymph nodes (LN) were enlarged	Radical nephrectomy with dissection LN stations	Alive and disease free at 13 months post-surgery.
Ghosh(2014) [5]	M	51	Dull and intermittent pain	A solitary mass in the lower pole of right kidney	Nephrectomy	Asymptomatic with no evidence of recurrent or metastatic disease 12 months after surgery
Tapan Kumar Sahoo (2015) [7]	F	50	pain on the right side of the abdomen for 6 months	mild to moderate enhancing mass of size approximately 6 × 8 cm at upper pole of the right kidney	Radical nephrectomy	on regular follow-up for 6 months without any evidence of disease.
Wang 2016 (et al) [6]	M	61	Lumbago and gross hematuria	Multiloculated cyst-like masses with soft tissues of right kidney and fuzzy perirenal fatty space	Radical nephrectomy	No febrile, gross hematuria or abnormal abdominal signs one month after surgery

Due to its nonspecific clinical presentation compared to other kidney tumors, hydronephrosis presents a great challenge for early diagnosis. The main symptoms presented by the five case reports of SCC of the rena parenchyma (Table 1) are hematuria and pain. Image examinations are valuable but are non-specific. Because of the rarity and the short follow-up of the cases, a prognosis is very difficult to predict.

Ultrasonography typically only reveals only hydronephrosis. Conversely, CT is an important tool and can provide high-resolution imaging to evaluate masses and stages in renal malignancies. In the present case, CECT revealed hydronephrosis with a markedly dilated cystic pelvicalyceal system with perinephric chronic inflammation. This is consistent with the renal specimen seen after resection. Ureteral obstruction is the main cause of the presenting symptoms.

Furthermore, the significance of the case can also be considered as a lesson, which teaches us to consider the possibility of renal malignant tumors when encountering the patients with long-term hydronephrosis, uneven thickness of kidney parenchyma and uneven enhancement of CT scanning.

According to the patient’s medical history and primary clinical examinations, tuberculosis and uretero pelvic junction obstruction were rather unlikely. One of the most consistent initial diagnoses is a neoplasm with unknown character. Primary SCC of the kidney should further rule out metastatic SCC with the combination of clinical history, imaging studies, and histopathology [7, 10]. Nevertheless, it is difficult to make a definite diagnosis prior to surgery considering the nonspecific features of this case. FDG-PET/CT has been validated as an effective tool in diagnosis, preoperative evaluation and prognosis of kidney malignancies [11]. However, FDG-PET/CT were ineffective for distinguishing renal inflammation diseases from various types of tumors in some cases [6]. Renal tumors can be biopsied by puncture. Patients with hydroneprosis can be biopsied through ureteroscopy for stenosis. In addition, urocytology is helpful to determine the diagnosis of primary or invading lesions in renal pelvis and calves.

Regrettably, this patient did not undergo any examinations other than those mentioned above. The main reasons for this were as follows: 1) The imagological diagnosis showed dilation of the collecting system and chronic perinephritis, and the judgment of the tumor was not clear. 2) The Double J stent implantation was performed in an outside hospital. Due to the two reasons, it was difficult to make decisions on puncture biopsy, ureteroscopy and biopsy of stenoses. In addition, the ureteroscopy procedure may have introduced an increased the risk of tumor metastasis. 3) The positive rate of urocytology was low and not beneficial in affecting the decision to perform surgery. Meanwhile the double J stent placed in the outside hospital had just been removed before admission. Therefore, infectious lesions were primarily considered, affecting the clinical decision. Afterwards, the tumor did not invade the collective system. 4) The most important factor was the refusal of the patient and her family to undergo further medical examination as we suggested, including FDG-PET/CT because of the cost. They insisted on proceeding directly to surgery. Therefore, after the informed consent was signed, retroperitoneal laparoscopic radical nephrectomy was performed.

Because renal SCC is insensitive to chemotherapeutics and radiotherapy, the patient did not receive any further treatment. The cost of treatment was also taken into consideration. In addition, the prognosis of renal SCC is generally poor, and more extensive follow-up data is essential to evaluate the prognosis of renal parenchyma SCC.

In conclusion, SCC of the kidney parenchyma is a rare upper tract urothelial carcinoma, which presents a diagnostic challenge to the urologist and which should be considered in the patients with chronic hydronephrosis. According to the recent literature, 5 cases of renal parenchyma SCC have been reported to date (Table 1). As more cases are presented in the literature, urologists will be able to make a quick and accurate diagnosis and provide treatment of this disease by understanding its presentation and natural history.

## Data Availability

The datasets used and/or analyzed during the current study are available from the corresponding author on reasonable request.

## Abbreviations

SCC: Squamous cell carcinoma
WBC: White blood cell count
PLT: Total number of platelets
ESR: Erythrocyte sedimentation rate
PCT: Procalcitonin
CECT: Contrast-enhanced computed tomography
PPD: Tuberculin purified protein derivative
CT: Computed tomography
GFR: Glomerular filtration rate
FDG-PET/CT: [18F]-fluorodeoxyglucose- positron emission tomography-computed tomography
